# Identifying educator behaviours for high quality verbal feedback in health professions education: literature review and expert refinement

**DOI:** 10.1186/s12909-016-0613-5

**Published:** 2016-03-22

**Authors:** Christina E. Johnson, Jennifer L. Keating, David J. Boud, Megan Dalton, Debra Kiegaldie, Margaret Hay, Barry McGrath, Wendy A. McKenzie, Kichu Balakrishnan R. Nair, Debra Nestel, Claire Palermo, Elizabeth K. Molloy

**Affiliations:** Health Professions Education and Educational Research (HealthPEER), Faculty of Medicine, Nursing and Health Sciences, Monash University, Melbourne, Australia; Department of Physiotherapy, Faculty of Medicine, Nursing and Health Sciences, Monash University, Melbourne, Australia; Centre for Research on Assessment and Digital Learning, Deakin University, Geelong, Australia; Faculty of Arts and Social Sciences, University of Technology Sydney, Ultimo, Australia; Institute of Work-Based Learning, Middlesex University, London, UK; School of Human, Health and Social Science, Central Queensland University, Rockhampton, Australia; Faculty of Health Science, Youth and Community Studies, Holmesglen Institute and Healthscope Hospitals, Holmesglen, Melbourne, Australia; Faculty of Medicine, Nursing and Health Sciences, Monash University, Melbourne, Australia; Faculty of Education, Monash University, Melbourne, Australia; School of Medicine and Public Health, Faculty of Health and Medicine, University of Newcastle, Newcastle, Australia; Department of Nutrition and Dietetics, Faculty of Medicine, Nursing and Health Sciences, Monash University, Melbourne, Australia; Monash Doctors Education, Monash Health, Monash Medical Centre, Clayton, Melbourne, Australia

**Keywords:** Feedback, Clinical practice, Delphi process, Health professions education, Educator behaviour

## Abstract

**Background:**

Health professions education is characterised by work-based learning and relies on effective verbal feedback. However the literature reports problems in feedback practice, including lack of both learner engagement and explicit strategies for improving performance. It is not clear what constitutes high quality, learner-centred feedback or how educators can promote it. We hoped to enhance feedback in clinical practice by distinguishing the elements of an educator’s role in feedback considered to influence learner outcomes, then develop descriptions of observable educator behaviours that exemplify them.

**Methods:**

An extensive literature review was conducted to identify i) information substantiating specific components of an educator’s role in feedback asserted to have an important influence on learner outcomes and ii) verbal feedback instruments in health professions education, that may describe important educator activities in effective feedback. This information was used to construct a list of elements thought to be important in effective feedback. Based on these elements, descriptions of observable educator behaviours that represent effective feedback were developed and refined during three rounds of a Delphi process and a face-to-face meeting with experts across the health professions and education.

**Results:**

The review identified more than 170 relevant articles (involving health professions, education, psychology and business literature) and ten verbal feedback instruments in health professions education (plus modified versions). Eighteen distinct elements of an educator’s role in effective feedback were delineated. Twenty five descriptions of educator behaviours that align with the elements were ratified by the expert panel.

**Conclusions:**

This research clarifies the distinct elements of an educator’s role in feedback considered to enhance learner outcomes. The corresponding set of observable educator behaviours aim to describe how an educator could engage, motivate and enable a learner to improve. This creates the foundation for developing a method to systematically evaluate the impact of verbal feedback on learner performance.

## Background

Health professions education is characterised by work-based learning where a student or junior clinician (a ‘learner’) learns from a senior clinician (an ‘educator’) through processes of modelling, explicit teaching, task repetition, and performance feedback [1, 2]. Feedback, which follows an educator observing a learner perform a clinical task, is an integral part of this education. This may occur ‘on the run’, during routine clinical practice or as scheduled feedback during workplace-based assessments, planned review sessions, or at mid- or end-of-attachment performance appraisals.

Feedback has been defined as a process in which learners seek to find out more about the similarities and differences between their performance and the target performance, so they can improve their work [3]. This definition focuses on the active role of the learner and highlights that feedback should impact on subsequent learner performance.

Feedback needs to help the learner develop a clear understanding of the target performance, how it differs from their current performance and what they can do to close the gap [4–6]. To accomplish this, a learner has to construct new understandings, and develop effective strategies to improve their performance. A learner also has to be motivated to devote their time and effort to implementing these plans, and to persist until they achieve the target performance.

In an attempt to enhance learner-centred feedback, it is enticing to focus on the learner and their role in the feedback exchange. However given that educators typically lead educational interactions, particularly in the early stages, targeting the educator’s role in feedback may have a greater influence in cultivating learner-centred feedback. A skilled educator can create an optimal learning environment that engages, motivates and supports learners, thereby enabling them to take an active role in evaluating their performance, setting valuable goals and devising effective strategies to improve their performance [7, 8]. Learners who have experienced such sessions could then carry forward a clear model of high quality feedback into future interactions throughout their professional life.

Experts in health professions education assert that feedback is a key element in developing expertise [6, 9–14]. Learners in the health professions also believe feedback can help them and they want it [15–18]. However there is limited evidence to support this conviction that feedback improves the performance of health professionals. The strongest evidence is from two meta-analyses, which indicated that audit followed by feedback improved adherence to clinical guidelines [19, 20]. Beyond the health professions there is stronger evidence. In a synthesis of 500 meta-analyses (180,000 studies), feedback was reported to have one of the most powerful influences on learning and achievement in schools [4]. Another meta-analysis of 131 studies compared feedback alone with no feedback on objective measures of performance of diverse tasks. That analysis also supported the conclusion that feedback improved performance [21].

Despite the enviable theoretical benefits of feedback, problems have been reported in practice. In observational studies of face-to-face feedback, educators often delivered a monologue of their conclusions and recommendations. Learners spoke little, asked few questions, minimised self-assessment (if asked) and were not involved in deciding what was talked about, explaining their perspective or planning ways to improve [22–27].

Observational studies and reviews of feedback forms indicated that educators’ comments were often not specific, did not identify what was done satisfactorily and what needed improvement, and did not include an improvement plan [23, 28–30].

Educators have reported that they did not feel confident in their feedback skills. In particular they avoided direct corrective comments as they feared it could undermine a learner’s self-esteem, trigger a defensive emotional response or spoil the learner-educator relationship. Educators experienced negative feelings themselves, such as feeling uncomfortable or mean [17, 22, 23, 31].

Feedback does not always improve performance and can even cause harm [4, 19, 20, 32, 33]. In Kluger and DeNisi’s meta-analysis [21], approximately a third of studies found that performance deteriorated following feedback.

Learners have reported that they do not always implement feedback advice. Their reasons included they did not consider there was a problem, did not believe the educator’s comments were credible or relevant [34, 35], or did not understand what needed improving or how to do it [34, 36]. Learners have also reported experiencing strong negative emotions such as anger, anxiety, shame, frustration and demotivation following feedback, especially if they thought feedback comments were unfair, derogatory, personal or unhelpful [17, 36–38].

Our goal is to promote high quality feedback by helping educators to refine the way they participate in feedback, and subsequently to enhance learner outcomes. It is not clear what comprises high quality, learner-centred feedback or how educators can promote it. [39, 40]. One explanation for the mismatch between the theoretical benefits of feedback and the problems experienced in practice, is that feedback involves multiple unidentified elements that may influence the outcome. Therefore it would be useful to clarify the components of an educator’s role in feedback required to achieve the aim of engaging, motivating and enabling a learner to improve their skills and develop a list of key educator behaviours that describe how these objectives could be accomplished in clinical practice.

In this study we chose to target the educator’s role first because educators have substantial influence and a primary responsibility to model high quality feedback skills. The setting we focused on was scheduled face-to-face verbal feedback following observation of a learner performing a task, as this is a particularly common form of feedback in the workplace education of health professionals.

## Methods

In this paper we describe the first phase in this process, which had two stages. The first stage involved conducting an extensive literature review to delineate the key elements of an educator’s role in effective feedback. In the second stage, a set of correlated educator behaviours was created and then refined in collaboration with an expert panel.

### Stage 1: literature review

The literature review was conducted to identify distinct elements of an educator’s role in feedback asserted to help a learner to improve their performance and the supporting evidence. The elements describe the key goals of an educator in high quality, learner centred feedback i.e., what needs to be achieved but not necessarily how to do it. In addition published instruments (or portions thereof) designed to assess face-to-face verbal feedback in health professions education were reviewed for descriptions of educator behaviours considered to be important in effective feedback.

**Fig. 1:**
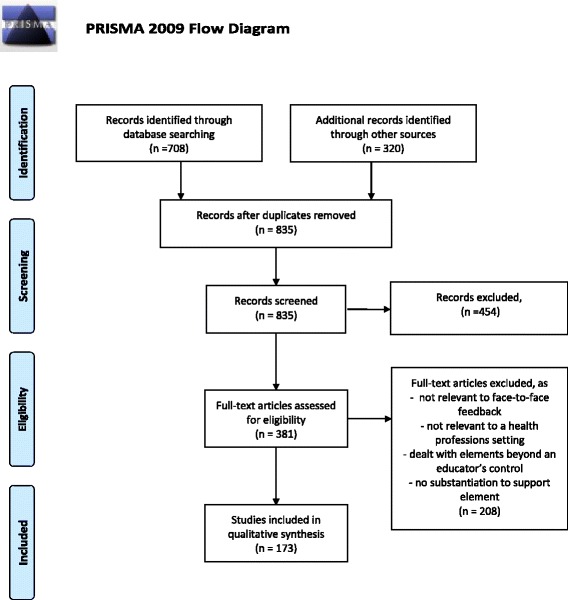
PRISMA flow diagram for the literature review

The target information was embedded within diverse articles spread across a broad literature base and was poorly identified by standardised database search terms. We therefore utilised a ‘snowball’ technique [13, 41]. This began with identifying systematic reviews on feedback plus published articles and book chapters in the health professions, education, psychology and business by prominent experts. When authors cited articles to support claims and recommendations, the original substantiating source was traced. Additional relevant articles were identified through bibliographies and citation tracking. This continued to the point of saturation where no new elements were identified. In addition, published instruments (or portion thereof) designed to assess face-to-face verbal feedback in health professions education were searched to identify relevant educator activities. Published literature was searched across the full holdings of Medline, Embase, CINAHL, PsychINFO and ERIC up to March 2013, and then continued to be scanned for previously unidentified elements until September 2015 (see Fig. 1).

#### Element construction

Elements were constructed by analysing and triangulating supporting information extracted during the literature review. Potential elements and substantiation were extracted by one researcher (CJ) and verified by core research team members (JK and EM). Similar elements were grouped and those with overlapping properties were collapsed. The core research team used an iterative process of thematic analysis [42] to develop a list of elements that described distinct aspects of an educator’s role in feedback.

### Stage 2: Development and refinement of the educator behaviour statements

The next step was to operationalise the elements by reconstructing them as statements describing observable educator behaviours that exemplify high quality feedback in clinical practice. An initial set of statements was developed by the core research team, using the same iterative process of thematic analysis, in accordance with the following criteria [43]: the statement describes an observable educator behaviour, that is considered important for effective feedback that results in improved learner performance, targets a single, distinct concept, and uses unambiguous language with self-evident meaning.

A Delphi technique was used to develop expert consensus on the statement set, in which sharing of anonymous survey responses enables consensus to develop as opinions converge over sequential rounds [44–46]. An expert panel was formed. All panel members provided informed consent. Members refined the individual statements and the composition of the list as a whole, and developed consensus on each statement (defined as over 70 % panel agreement) during three rounds using a Delphi technique [47].

#### Expert panel

**Fig. 2:**
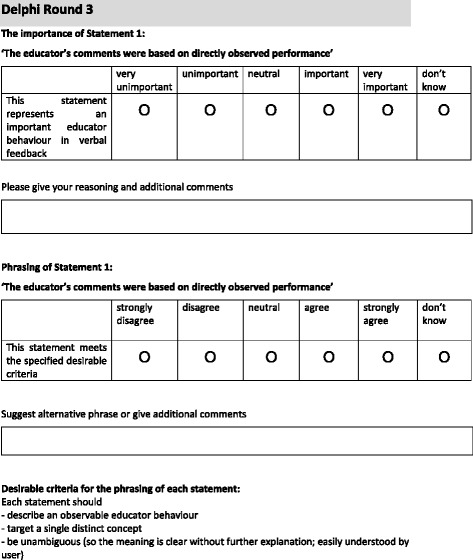
Desirable criteria and example of two questions from Delphi Round 3 survey

The research team invited nine Australian experts with experience in health professions education, feedback, psychology, education and instrument development to join research team members (JK and EM) to create a panel to refine the statement set. The primary researcher (CJ) acted as the facilitator. A structured survey presenting the initial statements was distributed to panel members using online survey software. For each statement, panel members were asked to consider two questions i) importance: ‘this statement represents an important educator behaviour in verbal feedback’ (rating options were ‘very unimportant, unimportant, neutral, important, very important or don’t know’) and ii) phrasing: ‘this statement meets the specified criteria’ (rating options were ‘agree, neutral, agree, strongly agree or don’t know’). For each question, panel members were asked to provide their reasoning and additional comments in free text boxes. Criteria for each statement and examples of two questions from the survey are presented in Fig. 2.

After each round, the ratings and comments were analysed using an iterative process of thematic analysis [42], and the educator behaviour statements refined accordingly. For the following round, a revised set of statements was circulated. This was accompanied by summarised anonymous panel responses from the previous round for participants to consider before continuing with the survey.

Following the conclusion of the three Delphi rounds, a face-to-face meeting of panel members was convened to resolve outstanding decisions. The meeting was audiotaped, transcribed verbatim and analysed using thematic analysis, and a set of educator behaviours was finalised.

Ethics approval for this study was obtained from Monash University Human Research Ethics Committee Project Number: CF13/1912-2013001005.

## Results

### Literature review

The database search identified a key set of reports [4, 10, 11, 13, 19–21, 48–54] that led to the identification of more than 170 relevant articles. These articles included observational studies of feedback, interviews and surveys of educators and learners, summaries of written feedback forms, feedback models, eminent expert commentary, consensus documents, systematic reviews and meta-analyses, and established theories across education, health professions education, psychology and business literature. There was little high quality evidence to clarify the effects of specific elements of feedback.

### Literature review: elements

**Fig. 3:**
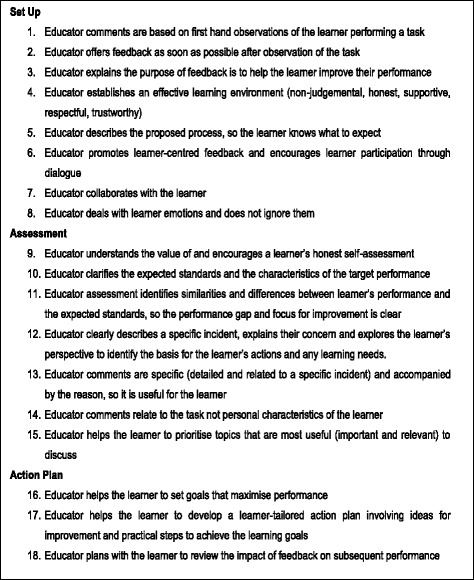
Key elements of an educator’s role in effective feedback, extracted and substantiated from the literature

Eighteen elements that describe the educator’s role in high quality feedback, were created by identifying substantiating information offered to support expert argument across diverse literature. These are presented in Fig. 3. The order is aligned to the usual flow of a feedback interaction including set up (including some elements that apply throughout), discussing the assessment and developing an action plan.

### Literature review: face-to-face verbal feedback instruments

The literature search identified 10 instruments (and additional modified versions) that, to some extent, assessed face-to-face verbal feedback in health professions education. It was hoped that these instruments would include items that described educator behaviours associated with effective feedback in clinical practice. However none of these instruments were designed to assess an educator’s contribution to an episode of face-to-face verbal feedback following observation of a learner performing a task in the workplace. Three instruments assessed a simulated patient’s feedback comments [55–59], three assessed an instructor’s debriefing to a group following a healthcare simulation scenario [60–62], two instruments assessed brief feedback associated with an Objective Structured Clinical Examination (in which the primary aim of the study was to determine if a senior medical student’s feedback was of a similar standard to a doctor’s) [63, 64], and two longitudinally assessed an educator’s overall clinical supervision skills, including feedback, across a clinical attachment [65–67].

### Development and refinement of the educator behaviour statements using a Delphi technique

#### Panel

All nine invited experts agreed to participate to create an eleven member panel; the primary researcher acted as facilitator. All panel members had senior education appointments at a hospital or university (the majority were professors and/or directors). The panel included seven health professionals (medicine, nursing, physiotherapy, dietetics and psychology) and several internationally recognised experts in feedback, education and training, simulation and instrument development. There was a high level of engagement by the panel throughout; all members completed each survey in full and made frequent, detailed additional comments.

#### Development of observable behaviour statements

The initial set of observable educator behaviours, developed by the core research team from the elements, contained 23 statements as some elements required more than one for operationalisation. This set was submitted to the Delphi process. After every round, the individual statements and the set as a whole were modified, based on the panel’s ratings and comments. Revisions included refining statements to better target the underlying concept, and rewording statements to better align with the specified criteria (see Fig. 1). Overlapping statements were combined and new ones were developed.

One example of how an element was refashioned into a corresponding observable educator behaviour, is described here. Element 4 states an “educator establishes an effective learning environment”. This was operationalised into “the educator showed respect and support for the learner” (Behaviour Statement 11) and “the educator indicated that while developing a skill, it is expected that some aspects can be improved and the educator is here to help, not criticise” (Behaviour Statement 4).

After completion of the third round, there were 25 statements in the set. Expert consensus was achieved for i) statement importance: all except one and ii) statement phrasing: all except three. These outstanding issues were resolved at the face-to-face panel meeting.

**Fig. 4:**
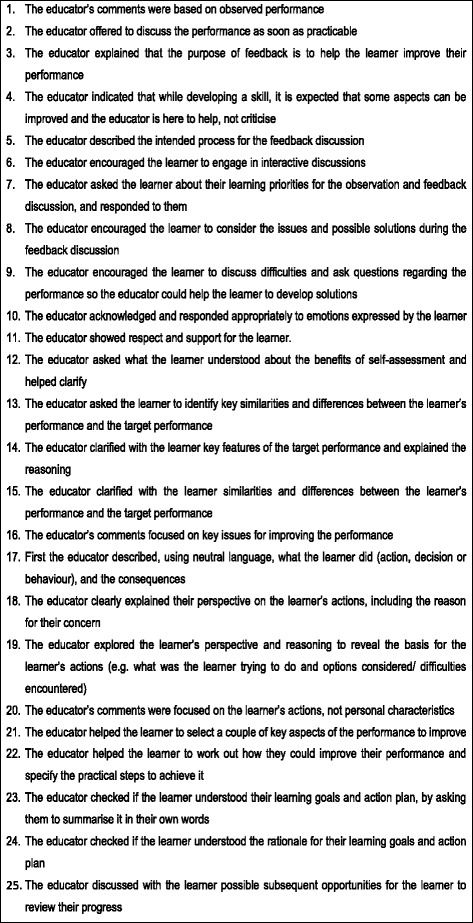
List of educator behaviours that demonstrate high quality verbal feedback in clinical practice

The final list, presented in Fig. 4, included 25 statements that explicitly describe observable educator behaviour in high quality verbal feedback.

## Discussion

We sought to distinguish the key elements of an educator’s role in feedback, endorsed by the literature, and to develop consensus on a set of observable behaviours that could engage, motivate and enable a learner to improve their performance in clinical practice. Support for these elements came from triangulating information from observational studies of feedback, surveys and interviews of educators and learners, summaries of written feedback forms, systematic reviews and meta-analyses of feedback, and established psychological and behavioural theories, in addition to expert argument, published across health professions, education, psychology and business literature. However there is little high quality evidence to substantiate these educator behaviours and they require formal testing to explore their impact in clinical practice. One of the drivers for this research was the desire to investigate whether specific constituents of feedback argued to be important, do indeed enhance learning.

### Characteristics of educator feedback behaviours in high quality feedback

We identified 18 distinct elements and 25 educator behaviours; this exposes the complexity of a feedback interaction. To facilitate further discussion and consideration, we propose four overarching themes that may describe the key concepts of high quality feedback.

1. The learner has to ‘do the learning’
  A learner needs to develop a clear vision of the target performance, how it differs from their performance and the practical steps they can take to improve their subsequent performance (Statements: 14–16, 22–24) [4, 5, 68]. This requires the learner to make sense of an educator’s comments, to compare the new information with their previous understanding of the issue and resolve gaps or discrepancies [14, 69, 70]. A learner has to actively construct their own understanding; an educator cannot deliver it ‘ready-made’ to them. Feedback is best done as soon as the learner and educator can engage after the performance (Statement: 2). A learner can only work on one or two changes at a time, in accordance with theories of cognitive load [71]. This would suggest that it is important to prioritise the most important and relevant issues (Statement: 21) [14, 24]. As feedback is an iterative process, the progress achieved (or difficulties encountered) after implementing the action plan should be reviewed (Statement: 25) [5, 14, 72].
  The primary purpose of the learner’s self-assessment is to develop their evaluative judgement, contributing to their self-regulatory skills (Statements: 7–8, 12–13) [73, 74]. The learner is positioned to take responsibility for their own learning. As they compare their performance to the target performance, it offers an opportunity for them to clarify their vision of the target performance (Statement: 14), calibrate their assessment to the educator’s assessment (Statement: 15), and highlight their priorities and ideas about how their performance could be improved (Statements: 7–8) [72].
  Once the learner is seen as ‘the enacter’ of feedback, the educator’s role becomes ‘the enabler’. The educator uses their expertise to discuss the performance gap, explore the learner’s perspective and reasoning, clarify misunderstandings, help to solve problems, offer guidance in setting priorities and effective goals, and suggest ideas for improvement (multiple statements).
2. The learner is autonomous
  High quality feedback supports a learner’s intrinsic motivation to develop their expertise and respects their autonomy [75]. It recognises that the learner decides which changes to make (if any) and how they will do this. Feedback information is only ‘effective’ if a learner choses to implement it. This is more likely to occur when a learner believes an educator’s comments are true and fair, and will help them to achieve their personal goals. This is more likely when an educator’s comments are based on specific first-hand observations (Statement: 1) as a starting point for an open-minded discussion with the learner about the reasons for their actions, and enables identification of learning needs (Statements: 17–19) [10, 76, 77]. An educator’s comments are best directed to actions that can be changed, not personal characteristics (Statement: 20), that is, ‘what the learner did, not what the learner is’ [10, 21, 77]. Comments that target a person’s sense of ‘self’ (including valued self-concepts like ‘being a health professional’) or general corrective comments, may stimulate strong defensive reactions, and do not appear to improve task performance [21, 37, 78, 79]. To support a learner’s intrinsic motivation, an educator should offer suggestions as opposed to giving directives, explain the reasons for their recommendations and help a learner to develop an action plan that aligns with their (often revised) goals, priorities and preferences (Statements: 7,14,18,22,24) [75, 80, 81].
3. The importance of the learner-educator relationship
  The learner-educator relationship strongly influences face-to-face feedback; the personal interaction can enrich or diminish the potential for learning [4, 8, 82]. During the encounter, a learner’s interpretation of the educator’s message is affected by their knowledge and experience of the educator. If a learner believes an educator has the learner’s ‘best interests at heart’, is respectful and honest, this creates a trusting relationship and an environment that supports learning (Statements: 3–4,11) [8]. This sense of trust, or psychological safety, encourages the learner to take a ‘learning focus’ not a ‘performance focus’, so the learner can concentrate on improving their skills, as opposed to trying to appear competent by covering up difficulties (Statement: 9) [14, 78, 83]. Performance evaluation often stimulates emotions [6]. An educator may help by responding to a learner’s emotions appropriately (Statement: 10) [84]. In addition an educator should aim for a feedback process that is transparent and therefore predictable, which may help a learner manage feelings of anxiety about what is likely to happen in the session (Statements: 5) [39, 85].
4. Collaboration
  Collaboration, through dialogue, is essential for high quality feedback (multiple items). The learner and educator work together, with the common aim of creating an individually-tailored action plan to help the learner improve. The behaviours specified in the items are designed to promote shared understanding and decision-making. Feedback is more than two separate contributions; each one seeks, responds to and builds on the other’s input. Face-to-face verbal feedback offers a unique opportunity for direct, immediate and flexible interaction. This makes it possible for a learner or educator to seek further information, clarify what was meant, raise different perspectives, debate the value of various options and modify proposals in response to the other’s comments. Collaboration optimises the potential for a fruitful outcome because insufficient information, misunderstandings and other obstacles to success can be dealt with during the discussion.

### Research strengths and limitations

This research has several strengths. It addresses an important gap in health professions education with a practice-orientated solution. The research design was systematic and rigorous, starting with an extensive literature search followed by expert scrutiny. The literature search continued to the point of saturation but we cannot be sure that all relevant information was assembled. Countering the potential for oversight was the in-depth scrutiny by experts in the health professions and education.

## Conclusion

Work-based learning in the health professions [86] relies on effective verbal feedback but problems with current feedback practice are common. This research advances the feedback literature by creating an endorsed, explicit and comprehensive set of educator behaviours intended to engage, motivate and support a learner during a feedback interaction. The recommended educator behaviours provide a platform for developing a method to systematically evaluate the impact of the verbal feedback on learner performance.

*Examples of survey format and responses are available from the first author on request.
